# Enhanced Supercapacitor Performance Based on CoAl Layered Double Hydroxide-Polyaniline Hybrid Electrodes Manufactured Using Hydrothermal-Electrodeposition Technology

**DOI:** 10.3390/molecules24050976

**Published:** 2019-03-10

**Authors:** Guoshen Yang, Takahiro Takei, Sayaka Yanagida, Nobuhiro Kumada

**Affiliations:** Center for Crystal Science and Technology, University of Yamanashi, 7-32 Miyamae, Kofu, Yamanashi 400-8511, Japan; g16dga02@yamanashi.ac.jp (G.Y.); syanagida@yamanashi.ac.jp (S.Y.); kumada@yamanashi.ac.jp (N.K.)

**Keywords:** CoAl layered double hydroxides, conductive polymer polyaniline, electrochemical properties, pseudocapacitor

## Abstract

Electrodes with nanosheet architectures can offer the possibility to achieve enhanced energy storage performance. Herein, we have designed and synthesized novel nanosheet structures of CoAl layered double hydroxide (LDH)-polyaniline (PANI) nanocomposite thin films by a hydrothermal-electrodeposition method. The molecular structure, crystal structure, morphology and chemical composition of the composites were characterized by FT-IR, XRD (SXRD), FESEM, and XPS, whereas their electrochemical properties were evaluated by cyclic voltammetry, electrochemical impedance spectroscopy and galvanostatic charge-discharge tests. Compared with the unmodified CoAl LDH, the CoAl LDH-PANI exhibits significantly improved the specific capacitance and cyclic stability. The composite exhibits a high specific capacitance of 528 F/g at a current density of 10 A/g and excellent cyclic stability with an increase of the specific capacitance of 42.7% after 6000 cycle tests. We revealed the degradation behavior of PANI in 1 M KOH/KCl electrolyte, and the active degradation products also further increased the total specific capacitance of the composite. The enhanced electrochemical performance of the nanocomposite can be attributed to its well-designed nanostructure and the synergistic effects of each component. By analyzing the band structure and density of states of CoAl LDH and PANI, we proposed the possible mechanism of synergistic effect in a new perspective.

## 1. Introduction

Supercapacitors (also known as electrochemical capacitors), as promising energy storage devices, have excellent electrochemical properties such as fast charge-discharge capability, high power density, and long cycle life [1,2,3]. Pseudocapacitors (also called Faraday capacitors) represent an appealing type of supercapacitors, which have the potential to achieve high specific capacitance and high energy storage resulting from the active electrode materials and reversible redox reactions [4,5,6]. The research on pseudocapacitors has therefore drawn increasing attention in recent years [7]. In the meantime, the energy stored in the supercapacitor is still relatively lower than battery and thus limiting its application in high cycle life and power density [8].

Electrode material is the most critical part of supercapacitor, and it is also a key factor in determining its performance [9]. At this stage, there are three main categories of electrode materials: transition metal oxides/hydroxides, carbon-based, and conductive polymers [10]. The transition metals, including layered double hydroxides (LDHs), are ideal electrode materials for pseudocapacitors owing to their high redox activity, structural controllability and environmentally friendly nature [11,12,13]. Nevertheless, the relatively poor cycling life and low electronic conductivity of the LDHs limit their practical applications. To further improve the electrochemical performance, several unique carbon hybrid materials such as activated carbon/LDHs, carbon nanotube/LDHs, graphene/LDHs, and graphene oxide/LDHs have been fabricated [14,15,16,17]. Recently, more advanced and innovative nanocomposites based on LDHs have been constructed to improve the performance of pseudocapacitive materials by creating nanostructures, such as hollow LDHs spheres, LDHs/metal hydroxides, and LDHs/conducting polymers hybrid materials [14,18,19,20,21,22,23]. A few studies so far have focused on LDHs/conducting polymers nanocomposites, with the aim of improving the specific capacitance and cyclic stability. Han et al. successfully prepared CoAl LDH@PEDOT nanocomposite, which exhibited excellent long-term cycling stability [23]. Whereas, its specific capacitance didn’t show a significant increase and needed to be further improved. Shao et al. prepared a PPy@LDH core-shell composite via a two-step electrosynthesis, which exhibited a high energy density and excellent cycling stability [24]. Compared to conductive polymer polypyrrole (PPY) and poly(3,4-ethylenedioxythiophene) (PEDOT), polyaniline (PANI) has broader application and becomes important research subject because of its excellent electrical conductivity, good environmental stability, high specific capacitance, ease of synthesis and redox stability [25,26,27,28]. Little is known, however, about the investigation of CoAl LDH-PANI nanocomposite. The aim of this research was consequently to study the performance of CoAl LDH-PANI nanocomposite. We deemed that the study of CoAl LDH-PANI would usefully supplement and extend the research field of LDHs/conducting polymer hybrid electrode.

Inspired by the idea that conductive polymer of PEDOT and PPY can improve the electrochemical performance of LDHs, here, we elaborately designed and fabricated CoAl LDH-PANI nanocomposites directly grown on a Ni substrate by the hydrothermal-electrodeposition route and used them a binder-free electrode for pseudocapacitor. The nanocomposite exhibited significantly improved specific capacitance, good rate performance, and cyclic stability. The synergistic effect of inner LDH and outer PANI coating layer was studied in detail. Hence, we also believe that this work provides a promising approach for design and fabricate LDH/PANI electrode, which can be potentially used in energy storage devices.

## 2. Results and Discussion

### 2.1. Structural Study

**Figure 1:**
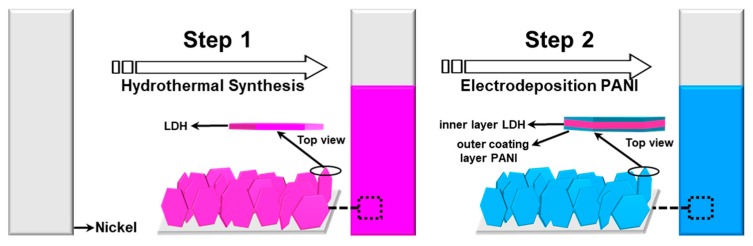
Schematic illustration of the two-step fabrication process of CoAl LDH-PANI nanosheet structures.

Our approach for constructing hierarchical CoAl LDH-PANI as a binder-free electrode involves a two-step process, as schematically shown in Figure 1. Firstly, vertically arranged CoAl LDH nanosheets were obtained on a Ni substrate as a binder-free electrode through homogeneous hydrothermal method. Secondly, a thin layer of conductive polymer PANI was hybridized with the pristine CoAl LDH by electrodeposition technique to form the inner/outer coating layer structures.

**Figure 2:**
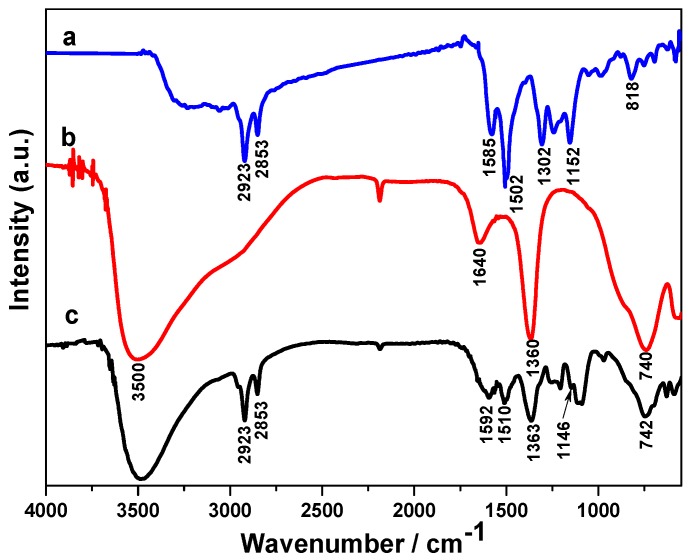
FT-IR spectra of (a) PANI, (b) CoAl LDH, and (c) CoAl LDH-PANI composite.

The molecular structures of as-prepared samples were characterized by Fourier transform infrared (FT-IR) spectroscopy. The FT-IR spectrum of pure PANI in Figure 2a shows the spectrum information is consistent with previously reported results [29,30,31,32]. The peaks at 2923 and 2853 cm^−1^ are separately ascribed to the asymmetric and symmetric stretching vibration of -CH_2_-, which caused by sodium dodecyl sulfate (SDS) doped with PANI [33]. The main peaks at 1585 and 1502 cm^−1^ are assigned to stretching deformations of benzene and quinoid rings. Also, the bands at 1302, 1152 and 818 cm^−1^ can be attributed to C-N stretch vibration, the aromatic C-H in the plane and out of plane bending vibration of the 1,4-disubstituted benzene ring, respectively. As to the CoAl LDH in Figure 2b, the strong, broad band around 3500 cm^−1^ can be explained as the metal-OH stretching mode and hydrogen bond interlayer H_2_O surrounding the interlayer anion [34]. The weak absorption band near 1640 cm^−1^ can be assigned to the H-O-H bending vibration of interlayer water molecules. The intense peaks at 1360 and 740 cm^−1^ can be attributed to CO_3_^2−^, owing to the asymmetric stretching vibration of the C-O bond. The lower wavenumber absorption bands at 400–700 cm^−1^ belong to the M-O, O-M-O, and M-O-M related vibrational modes of LDHs [9]. After the PANI modified CoAl LDH, several new characteristic peaks appear at 2923, 2853, 1592 and 1510 cm^−1^; these peaks were associated with PANI-SDS, which indicated the CoAl LDH has been successfully coated by PANI.

**Figure 3:**
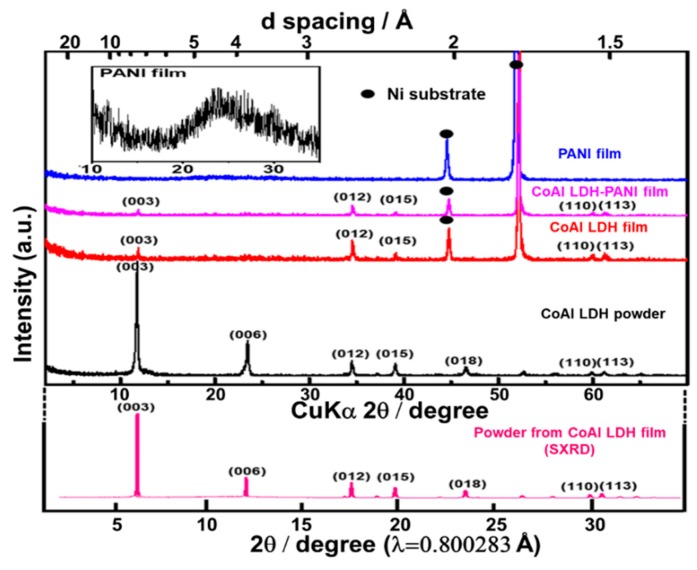
XRD patterns of CoAl LDH powder, CoAl LDH film, CoAl LDH-PANI film, PANI film, and SXRD patterns of the powder obtained from CoAl LDH film.

As shown in Figure 3, the crystal phase of each sample is further confirmed by X-ray diffraction (XRD). For the CoAl LDH powder, the diffraction peaks at 2θ values of around 11.5°, 23.1°, 34.3°, 38.9°, 46.4°, 59.7° and 60.9°, corresponding to (003), (006), (012), (015), (018), (110) and (113) of CoAl LDH phase (JCPDS: 51-0045), respectively [35]. Compared to the CoAl LDH powder sample, the diffraction peaks (00*l*) diminished for the thin film sample. Such a difference may result from a preferential orientation of LDH crystallites with their ab plane perpendicular to the Ni substrate, which can also be further confirmed by the FESEM observation. The existence of the LDHs on the substrate surface was also identified by SXRD. By calculating the interplanar spacing of the SXRD spectrum of CoAl LDH film, the results exhibited that the diffraction peaks of CoAl LDH film were consistent with CoAl LDH powder. It indicated that LDHs could grow well on the substrate by the hydrothermal method. Additionally, the presence of PANI in the CoAl LDH-PANI was also tested by XRD analysis. As the PANI is a long-range disordered amorphous structure, only one broad peak reflections around 2θ = 24.2° can be observed, which is caused by the emeraldine base form of PANI [36,37]. The XRD patterns of CoAl LDH-PANI film showed similar peaks to those CoAl LDH film, except that the peak intensities were decreased obviously. It could be attributed to the presence of the uniform coating of PANI on the CoAl LDH nanosheets.

**Figure 4:**
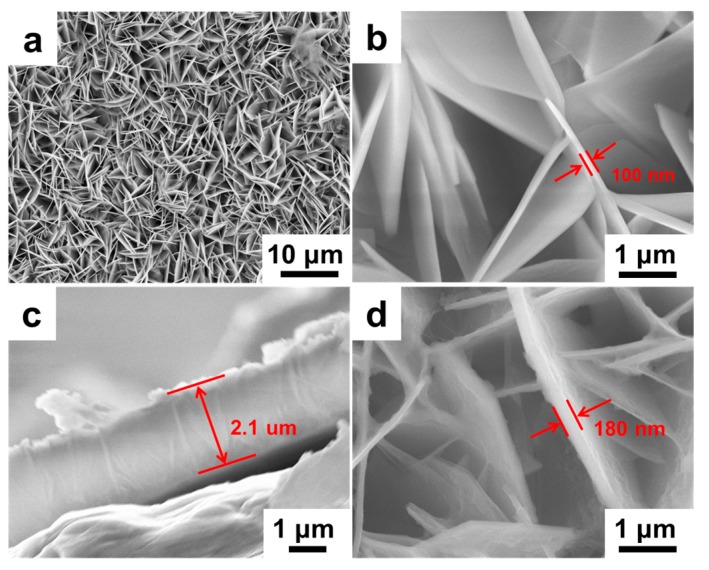
FESEM micrographs of (**a**,**b**) top-view of CoAl LDH, (**c**) cross-section of CoAl LDH, and (**d**) CoAl LDH-PANI composite.

The morphologies of CoAl LDH and CoAl LDH-PANI are investigated by field emission scanning electron microscope (FESEM), as shown in Figure 4. Figure 4a,b display top-view FESEM observations of the CoAl LDH nanosheets with a porous structure, and the nanoflakes with the thickness of ~100 nm. Figure 4a also displays that the CoAl LDH film can distribute uniformly on the substrate surface and consists of closely packed nanosheets with vertically arranged on the substrate in large amounts. A representative cross-sectional FESEM micrograph in Figure 4c shows that the CoAl LDH nanosheets have an average lateral size of ~2.1 μm. As to the composite in Figure 4d, it reveals that the LDH nanosheets are evenly wrapping with PANI coating layer and the morphology of CoAl LDH is well retained after the deposition of PANI, with the thickness of ~180 nm. 

In addition, the morphologies of CoAl LDH-PANI composite obtained at different electrodeposition time were further observed by FESEM (Figure S2). In Figure S2a, the morphology of PANI film is flat and smooth wrapping on the Ni substrate surface, which implies the chronoamperometry technique is appropriate to prepare CoAl LDH hybrid PANI. From Figure S2b–e, with increasing the deposition time from 0 s to 200 s, the PANI coating is deposited and tends to form a thin film on the surface of LDH nanosheets, which is uniformly covered the layer of thin film at deposition 200 s. Further prolonging the deposition time from 300 to 500 s, as shown in Figure S2f–i, the mass of PANI coating is further increased. Also, the gaps between the layers are gradually covered, and the porous structure is gradually blocked. It is known that the suitable mesopore size distribution is beneficial for the insertion of a large number of guest ions, which can effectively increase the storage capacity. It is also one of the vital factors to consider for electrodeposited CoAl LDH-PANI electrode.

**Figure 5:**
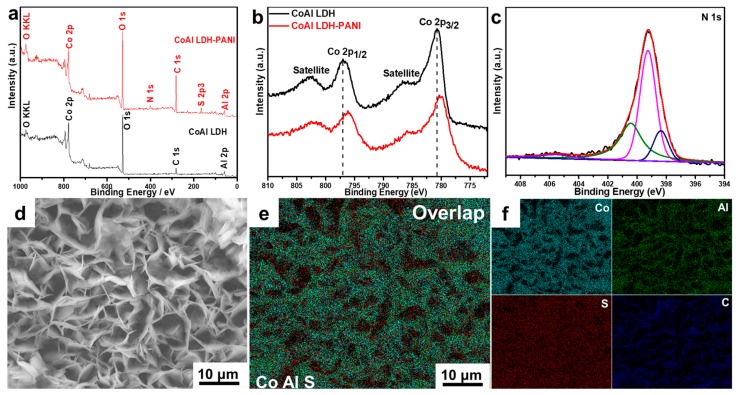
(**a**) The XPS full spectra and (**b**) Co 2p spectra of CoAl LDH and CoAl LDH-PANI. (**c**) N 1s spectrum of CoAl LDH-PANI. (**d**–**f**) FESEM image of CoAl LDH-PANI corresponding to the EDX elemental mapping images of Co, Al, S, and C showing uniform distribution of the elements.

In order to further study the elemental valence state and elemental distribution, X-ray photoelectron spectroscopy (XPS) and energy dispersive X-ray spectrometry (EDX) were carried out to characterize the pristine CoAl LDH and CoAl LDH-PANI samples. As illustrated in Figure 5a, Co 2p, O 1s, C 1s, and Al 2p peaks appear in the survey spectrum of pristine CoAl LDH, suggesting stacked CO_3_^2−^-LDH in CoAl-LDH. The full XPS spectrum of the CoAl LDH is also consistent with previously reported [38], while the presence of the N 1s and S 2p peaks in CoAl LDH-PANI spectrum indicate that CoAl LDH successfully hybrid PANI. Furthermore, S 2p peak revealed in the spectrum of the CoAl LDH-PANI, which was ascribed to the SDS doped PANI in the CoAl LDH-PANI composites. As shown in Figure 5b, the Co 2p line of CoAl LDH is split into Co 2p_1/2_ (796.9 eV) and Co 2p_3/2_ (780.6 eV) peaks accompanied by satellite bands. After hybrid with PANI, the Co 2p_3/2_ and Co 2p_1/2_ main peaks slightly shift to lower energy levels (796.1 and 780.1 eV, respectively). The shift in the binding energy of Co 2p peak position provides evidence of an interaction between the inner CoAl LDH and outer PANI coating layer. The N 1s line of CoAl LDH-PANI in Figure 5c can be deconvoluted into three peaks at 398.50, 399.27 and 400.37, as reported previously [32]. EDX analysis in Figure 5d–f shows homogeneously distributed elements, Co, Al, C, and S, which implied the CoAl LDH-PANI was well distributed on the substrate surfaces. As the low energy of the characteristic X-rays in light elements, the N element has not been captured. In additional, the superposition image in Figure 5e reveals that S element is uniformly decorated on the nanosheets and it gives visualized evidence that PANI is evenly coated the CoAl LDH nanosheet, resulting in uniform CoAl LDH-PANI inner/outer coating nanostructure.

### 2.2. Electrochemical Performance

**Figure 6:**
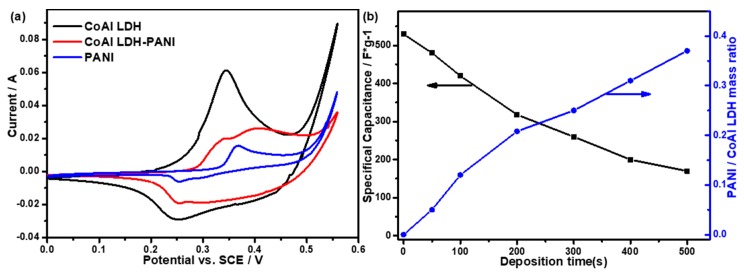
(**a**) CV curves of pristine PANI, CoAl LDH, and CoAl LDH-PANI composite obtained by deposition 200 s. (**b**) Specific capacitance values (obtained from CV measurement) and mass ratio of PANI: LDH for CoAl LDH-PANI electrode as a function of deposition time. (scan rate 20 mV/s)

Cyclic voltammetry (CV) was carried out to study the electrochemical performance of as-prepared electrodes using a three-electrode system at a scan rate of 20 mV/s. For the PANI electrode in Figure 6a, the CV curve possesses a pair of redox peaks (0.37/0.26 V), which has been reported previously [39]. As to the CoAl LDH electrode, the CV curve possesses a pair of redox peaks (0.34/0.25 V), which is caused by the quasi-reversible Faradic redox reaction (1) [9]:

(1) $$\mathrm{Co}{\left(\mathrm{OH}\right)}_{2}+{\mathrm{OH}}^{-}↔\mathrm{CoOOH}+H_{2}O+e^{-}$$

The nonrectangular shape of the CV curve also indicated that Faradaic reactions contributed to most of the charge storage.

For the CoAl LDH-PANI electrode, the CV curve possesses characteristic redox peaks of CoAl LDH (0.34/0.25 V) and PANI (0.39/0.29 V), which revealed the composite possesses the characteristics of both constituents. Unexpectedly, the CoAl LDH-PANI exhibited a lower current than those of the CoAl LDH. For the composite, the intensity of the CV curve was weakened, and the peak potential has slightly changed, which was caused by hybrid PANI in the initial cycle. The pristine PANI electrode exhibited a very lower resulting current than those of CoAl LDH and CoAl LDH-PANI electrodes. From the data in Figure 6b, the specific capacitance values of CoAl LDH-PANI are a trend of decreasing with the electrodeposition time increases. Comprehensive analysis of the morphologies in Figure 5 and specific capacitance values in Figure 6b, the CoAl LDH-PANI with deposition 200 s (PANI: CoAl LDH mass ratio of 0.21:1) was chosen as the test electrode for further evaluation of electrochemical performance. By comparing the electrochemically active Co (II) amount in the both pristine CoAl LDH and composite electrodes, it indicated that the inner active CoAl LDH in the composite have not been fully utilized at the initial cycle (See supporting information for detail). Figure S3a,c show CV curves of the pristine CoAl LDH and CoAl LDH-PANI electrodes at various scan rates. As the scan rate increases, the cathodic and anodic peaks shift to lower and higher potentials, respectively. And this shift was caused by the polarization of the electrode in highly porous LDH. As shown in the insets of Figure S3b,d, the almost linear relationship of the plot of anodic peak current versus the scan rate displays the surface-controlled redox reaction, which indicated the pseudocapacitance behavior of both electrodes [40]. As to the pseudocapacitance-battery behaviors of CoAl LDH and CoAl LDH-PANI, the electrochemical performances of the electrodes were assessed by determining the specific capacitance (F/g) and specific capacity (mAh/g), respectively.

Rate capability is one of the significant parameters for supercapacitor. Herein, galvanostatic charge-discharge (GCD) curves were used to test the rate capability of the pristine CoAl LDH and CoAl LDH-PANI electrodes (Figure S4). Figure S4 illustrates galvanostatic discharge curves at various current densities in the initial cycle numbers. At the same current density, the specific capacitance of CoAl LDH-PANI electrode was lower than that of CoAl LDH electrode. The specific capacitance of both electrodes has a high retention rate at high current density. At a high current density of 40 A/g, the specific capacitance maintains 70.5% and 67.7% for the pristine CoAl LDH and CoAl LDH PANI electrodes, which revealed good rate capability of the as-prepared electrode. The GCD test results indicated that the composite electrode also has good rate performance. 

In the initial cycle, the reduced capacitance of the composite has further analyzed the reasons by the electrochemical impedance spectroscopy (EIS) tests. Figure S5 shows the Nyquist plots of the EIS spectra for CoAl LDH and CoAl LDH-PANI, which consist of approximate semi-circles in the high-frequency range and an inclined straight line in the low-frequency range. The Nyquist plots are analyzed by applying the equivalent circuit shown in the inset of Figure S5. A summary of the CoAl LDH and CoAl LDH-PANI fitting parameters from the impedance spectra is presented in Table S1. In the high-frequency range, the intersecting point with the real axis represents the equivalent series resistance (R_e_). In addition, the approximate semi-circle is associated with Faradic reactions, and its diameter represents the interfacial charge-transfer resistance (R_ct_). In the low-frequency range, the slope is caused by the Warburg impedance (W), which is related to the diffusion resistance of the OH^-^ electrolyte ions in the electrode pores [23]. Furthermore, in the very low-frequency range (≤ 100 mHZ), the EIS spectra deviate from the idealized porous electrode model, and the slope of the straight line is larger than 45°. For the PANI-coated CoAl LDH electrode, this deviation behavior becomes more pronounced. Such behavior at very low frequency, also reported by Cooper [41] and Chamaani [42], is related to the frequency dispersion originating from the deficiency in the porous electrode structures. As shown in the inset of Figure S5, the value of the intersecting point with the real axis of CoAl LDH-PANI (0.375 Ω) is slightly larger than that of CoAl LDH (0.304 Ω), revealing that PANI only a small increase resistance to the composite electrode. The diameter of the approximate semi-circles of CoAl LDH-PANI is larger than CoAl LDH, and the slope of the straight line for CoAl LDH-PANI is less than CoAl LDH. It indicated that the composite electrode has a larger interfacial charge-transfer resistance and higher diffusion resistances that correspond to a reduced capacitance, compared to CoAl LDH. To put that another way, the PANI-coated CoAl LDH leads to an increase in “inactive” material (inner CoAl LDH) which is not very accessible to the OH^−^ electrolyte ions, and thus causes lower utilization of active material in the initial cycle. The n-value obtained from the Nyquist plot fitting is between 0.7 and 1, indicating the capacitive behavior of the CoAl LDH and CoAl LDH-PANI electrodes.

**Figure 7:**
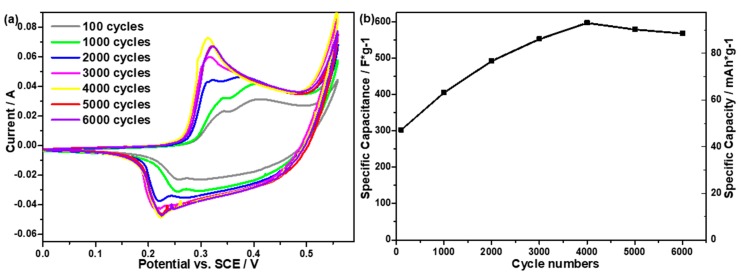
(**a**) CV curves of CoAl LDH-PANI electrode at different scan cycles. (**b**) Specific capacitance and specific capacity values (obtained from CV curves) of CoAl LDH-PANI electrode as a function of cycle numbers. (scan rate 20 mV/s).

The CV curves in Figure 7a exhibit the redox process of CoAl LDH-PANI electrode with cycle number from 100 to 6000 cycles. Interestingly, with the scan cycles increase, the CV curve tends to enlarge area accordingly, which indicates the specific capacitance and specific capacity increase correspondingly. As can be seen, the specific capacitances of CoAl LDH-PANI are calculated to be 308 F/g and 567 F/g after 100 and 6000 cycles. Besides, the specific capacity is also presented in Figure 7b. The specific capacity values of the CoAl LDH-PANI are calculated to be 47.9, 63.1, 76.6, 86.2, 92.9, 90.1 and 88.2 mAh/g as scan cycles of 100, 1000, 2000, 3000, 4000, 5000 and 6000 cycles, respectively. The CoAl LDH-PANI electrode exhibited increase current of redox peaks before 2000 cycles. The enhancement in specific capacitance could be attributed to the self-activation process of the inner CoAl LDH, which has also been supported by earlier reported [24,43,44]. Based on the EIS analysis, after hybrid with PANI, the inner active materials of CoAl LDH have not been fully utilized in the initial cycle. After repetitive redox process, the gradual activation of inner active points of the electrode materials exposed to the electrolyte, and hence enhanced the specific capacitance. It is also interesting to find that the current for the CoAl LDH-PANI increases continuously over 2000 cycles, with the disappearance of the PANI redox peak. It may have occurred the decomposition of polyaniline and formation of new active materials with highly electrochemically during the long scan process [45,46]. Perhaps the self-activation and degradation processes occurred simultaneously throughout the cycles. This enhanced capacitance is what we expect and will be further discussed the reasons below. 

**Figure 8:**
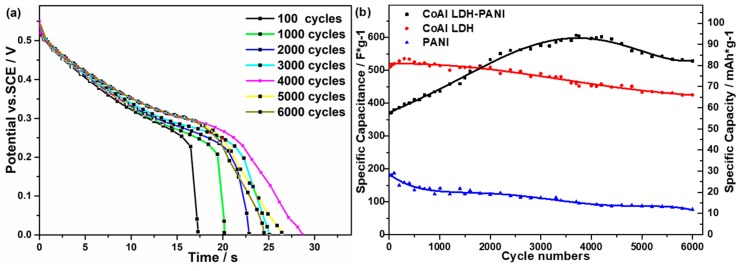
(**a**) Galvanostatic discharge curves of the CoAl LDH-PANI electrode at different scan cycles. (**b**) Cycling performance of the CoAl LDH, PANI, and CoAl LDH-PANI electrodes, respectively. (Current density 10 A/g)

The cycling performance is one of the most critical indices in the practical use of electrochemical supercapacitors. The long-term stability of as-prepared electrodes was tested by GCD, and the results are presented in Figure 8. As can be seen from Figure 8a, the galvanostatic discharge curves of the CoAl LDH-PANI electrode are extended the time with cycle numbers from 100 to 6000 cycles, which indicate the specific capacitance rise correspondingly. For the CoAl LDH electrode, the GCD time of the last 8 cycles decreased notably than that of the first 8 cycles (Figure S6). However, for the CoAl LDH-PANI, the GCD time of the last 8 cycles increased greatly compared with the first 8 cycles after 6000 GCD cycles. The results of this study indicated that the advantage of CoAl LDH-PANI was the increase of specific capacitance at high cycles. For the CoAl LDH electrode in Figure 8b, its specific capacitance increases from 510 to 535 F/g (79.3 to 83.2 mAh/g) in the initial 400 cycles due to the self-activation process, and then continuous decline from 535 F/g to 425 F/g (83.2 to 66.1 mAh/g, 83.3% retention after 6000 cycles). For the CoAl LDH-PANI electrode, it is pleasantly surprising to find that the specific capacitance increases from 370 to 600 F/g (57.6 to 93.3 mAh/g) over 3500 cycles, and then gradually decreased from 600 to 528 F/g (93.3 to 82.1 mAh/g, 142.7% retention after 6000 cycles). The cycling stability behavior was also observed for the PANI electrode. The specific capacitance of pristine PANI electrode has a dramatic drop from 180 to 76 F/g (28 to 11.8 mAh/g, 42.2% retention after 6000 cycles), which was ascribed to PANI structures suffer from a large volumetric alternation during the charge-discharge process. 

After comprehensive analysis of Figure 7 and Figure 8, the enhanced specific capacitance of CoAl LDH-PANI electrode may be attributed to both the self-activation process of electrode materials and decomposition of PANI to form new active substances during the long cycle process. Nevertheless, so far, the process of polyaniline degradation to form new active substances has not been made clear yet. In order to deeply investigate the reasons, we studied this reason by using the FT-IR, FESEM and XPS methods.

**Figure 9:**
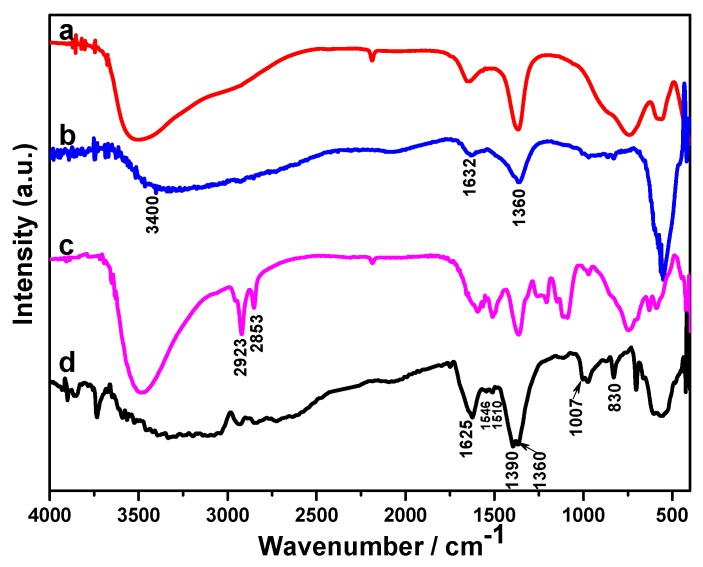
FT-IR spectra of (a) CoAl LDH obtained by pristine sample, (b) CoAl LDH obtained by 6000 GCD cycles, (c) CoAl LDH-PANI obtained by pristine sample, and (d) CoAl LDH-PANI obtained by 6000 GCD cycles.

After 6000 GCD cycles, the molecular structures of CoAl LDH and CoAl LDH-PANI samples were further characterized by FT-IR spectroscopy. What stands out in Figure 9 is the apparently different infrared spectrum of CoAl LDH-PANI before and after 6000 GCD cycles. For the CoAl LDH in Figure 9b, the intensity of the peak around 3400 cm^−1^ decreases and the peak becomes broader. The lower wavenumber absorption bands at 700–800 cm^−1^ become very weak. Combined with the analysis of Figure 2b, it implied that the part of CO_3_^2−^ anion interlayer space exchange to OH^−^. As to the CoAl LDH-PANI in Figure 9d, there are numerous new peaks appeared in the spectra, which indicate the oxidative degradation of PANI in alkaline solution. The peaks at 2923 and 2853 cm^−1^ disappear, which is assigned to the de-doping of SDS of PANI backbone in alkaline solutions (Possible degradation process sees the Supporting Information, Scheme S1). The release of dopant anion in the PANI backbone may cause its conductivity to decrease. Moreover, the absorption peaks of the degradation products group overlap with CoAl LDH, resulting in broader and enhanced absorption bands. The enhanced peak around 1625 cm^−1^ is caused by the H-O-H bending vibration of LDH interlayer water molecules, the stretching vibration of the terminal group C=O, and C=N stretching vibration of aromatic [47]. The weak band at 1546 cm^−1^ is assigned to the asymmetric stretching vibrations of NO_2_. The band at 1510 cm^−1^ becomes very weak in the spectrum, indicating the absence of p-phenylenediamine form (N-B-N) during the degradation process. The enhanced peak around 1390 cm^−1^ may be assigned to the interactions between the asymmetric stretching vibration of the C-O bond and symmetric stretching modes of the terminal group NO_2_. Herein, we speculated that the nitro group might be due to oxidizing the amino group and the imino group to form, which can be further confirmed by the XPS analysis. The bands at 1007 and 830 cm^−1^ are severally due to the C-H in-plane bending and the C-H out-of-plane bending vibrations of the 1,4-disubstituted benzene ring [31]. Considering the fact that the oxidative degradation of PANI, we may conclude that the polymer suffers oxidative degradation was ascribed by an inherent instability of PANI at high potentials. 

**Figure 10:**
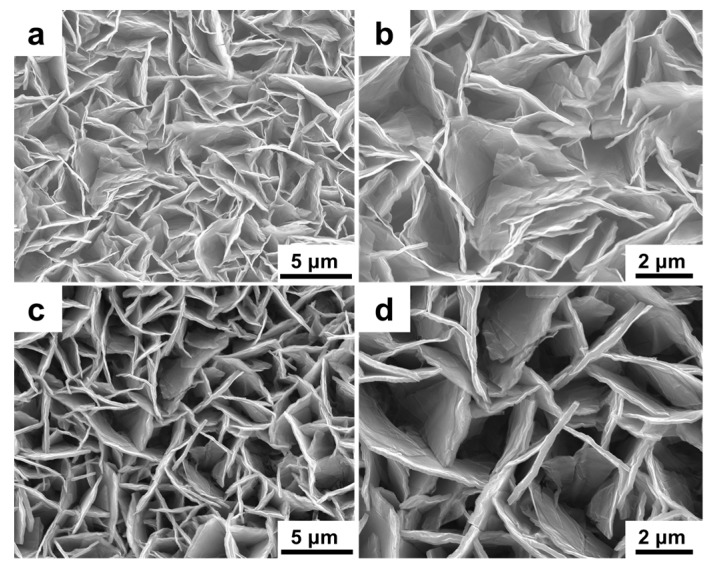
FESEM micrographs of (**a**,**b**) CoAl LDH and (**c**,**d**) CoAl LDH-PANI obtained by 6000 GCD cycles.

The morphologies of CoAl LDH and CoAl LDH-PANI electrodes were further observed by FESEM after 6000 GCD cycles. Compared with the pristine CoAl LDH (Figure 4a) vertically arranged on the substrate, it can be seen from the micrographs in Figure 10a,b that the nanosheets of CoAl LDH seem to have a slant angle to the substrate and partial collapse after 6000 GCD cycles. As to the CoAl LDH-PANI electrode in Figure 10c,d, they give visible evidence that the structures were well-preserved without any nanosheets cracks and collapses occurred. As we mentioned before, for the composite, the LDH nanosheets were uniformly wrapping with PANI coating layer and covered the gaps between the layers, which might play an essential role in the prevention of the electrolyte ions from degrading the inner nanosheets. Judging from this aspect, the presence of PANI coating layer sharply enhanced the structural stability of LDH nanosheets during the redox reaction. This good structural stability may be a direct answer for the better cycling stability. Figure S8 shows Co 2p spectra of pristine CoAl LDH-PANI and CoAl LDH-PANI obtained by 6000 GCD cycles. After 6000 GCD cycles, the Co 2p_3/2_ and Co 2p_1/2_ main peaks slightly shifted to higher energy levels with the intensity of the Co 2p peaks and satellite bands significantly decreased. These differences indicated that part of Co^2+^ in the composite was oxidized to Co^3+^ after 6000 GCD cycles. Furthermore, after 6000 GCD tests, the crystallinity seemed to be preserved due to the morphology of nanosheet on the plate could be observed in Figure 10. Given the result of SEM micrograph and XPS spectrum, it indicated that crystallinity of CoAl LDH in composite after 6000 GCD cycles was partially preserved.

**Figure 11:**
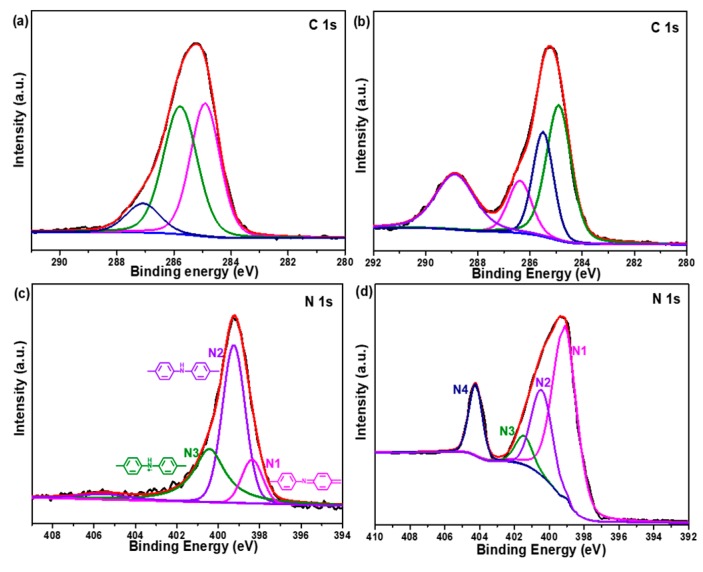
C 1s and N 1s spectra of (**a**,**c**) pristine CoAl LDH-PANI and (**b**,**d**) CoAl LDH-PANI obtained by 6000 GCD cycles.

Figure 11 illustrates the surface elemental C 1s and N 1s spectra of pristine CoAl LDH-PANI and CoAl LDH-PANI obtained by 6000 GCD cycles. The C 1s line of pristine CoAl LDH-PANI in Figure 11a can be deconvoluted in three peaks at different binding energies: 284.89 eV (C-C/C-H), 285.78 eV (C-N/C=N) and 287.12eV (C-O/C=O) [48]. The peak at 287.12 eV can be assigned to carboxylate carbon, indicating that CoAl-LDHs are partly intercalated by CO_3_^2−^ anion [49]. Whereas the C 1s line of CoAl LDH-PANI in Figure 11b can be deconvoluted in four peaks at different binding energies: 284.91 eV (C-C/C-H), 285.52 eV (C-N/C=N), 286.39 eV (C-O) and 288.89 eV (C=O). As mentioned in Figure 9, most of the carbonate ions in the LDH intercalation are replaced by hydroxide ions after LDH 6000 GCD cycles. Thus the C=O functional groups might be assigned to the formation of the terminal group C=O structure with degradation products. The N 1s line of pristine CoAl LDH in Figure 11c can be deconvoluted into three peaks at different binding energies: 398.50 eV (-N=), 399.27 eV (-NH-) and 400.37 eV (-N^+^H-) [39]. However, as to the CoAl LDH-PANI in Figure 11d, there is a new N 1s peak line appeared at 404.41eV (N4), which can be assigned to the formation of the terminal nitro group (-NO_2_) with degradation products [50,51]. And the N 1s peak also has similar deconvoluted with those of pristine CoAl LDH-PANI. Furthermore, the intensity of N1 line was maximum in Figure 11d, which indicated the composite has a higher ratio of quinoid groups. The above analysis results are also in accord with the FTIR analysis in Figure 9. The degradation products may be the critical factor for achieving higher electrochemical activity in electrolyte to increase the capacitance. Generally, hydroquinone can be oxidized to benzoquinone at 0.7 V versus SHE in acidic condition. This potential will decrease to around −0.1 V in the alkaline condition from Lewis equation. For the C=O group in our sample, the quinone ring is bonded with imino group which shows electron attraction by mesomeric (-M) effect. The imino group is connected with nitrophenol having the electron attractive property. These electron attractive groups will tend to increase the potential of the redox reaction between C=O and C-OH. Therefore, the redox process may occur within the potential window between 0 to 0.56 V versus SCE and contribute to increasing the capacitance. Furthermore, the degradation products of the terminal nitro group may also be closely related to achieving high electrochemical activity. Given the results of FT-IR and XPS analysis, it was also worthwhile mentioning that the degradation behavior of PANI was a difference between the alkaline and acid electrolyte solutions [47,52,53]. We were also aware that our research might also have the limitation. It is generally known that the random chain scission of polymer molecular chains is relatively complicated during the degradation of the PANI. Herein, we have only confirmed the possible terminal group structures generated during the degradation process. Regrettably, however, we were unable to definitely give the specific molecular formula of the new active materials.

**Figure 12:**
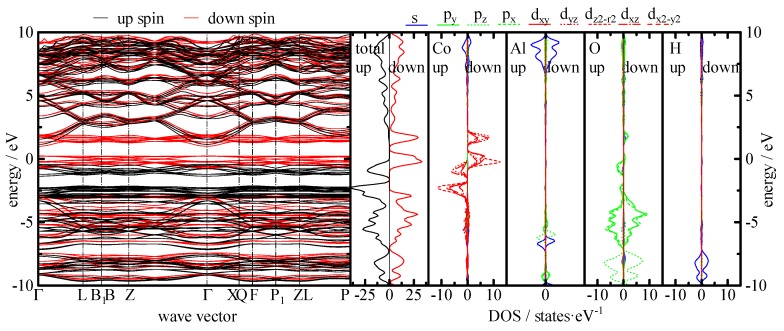
Band structure, total DOS, and partial DOS for each element of CoAl LDH.

Up to this point, a fundamental understanding of the synergistic mechanism of inner CoAl LDH and outer PANI layer is still missing. Here, we tried to reveal the synergistic mechanism from the perspective of the band structure. Figure 12 shows the band dispersion, density of states (DOS) of a total, Co, Al, and O calculated by ab-initio simulation. For these curves, the Fermi level is at 0 eV. From the band dispersion curve, Fermi level was slightly lower than the conduction band minimum (CBM), which was composed of down spin. Actually, CoAl LDH shows a slight conductive property. From the partial DOS, only the 3d electrons in Co was split between up and down spin nearby the Fermi level by exchange splitting due to delocalization of d orbital. In both spins, d orbitals are a bimodal shape with separation of around 1.9 eV, and components of each band are same as follows. The lower band is composed of large d*_z2-r2_* orbital and small amounts of d*_x2_*_-*y2*_, d*_xy_*, d*_yz_*, and d*_xz_* orbitals. In the higher band, large d*_yz_* and d*_xz_* orbitals and small d*_x2_*_-*y2*_ and d*_xy_* orbitals exist. These components implied that these separations were provided from degeneracy by crystal field splitting, focusing on the same direction of z-axis between electron orbital crystal structure. Since CoO_6_ octahedra are connected with edge sharing, d orbitals may be overlapped and provide electron conductivity with layer direction. On the other hand, the band with lower energy composed of O 2p is also split to p*_z_* and the others. The p*_z_* orbital binds with a 1s electron in a proton. There is a small energy gap of 0.25 eV at around 7–8 eV. Therefore, the electron should be hopped when the redox reaction (1) occurs. In the case of CoAl-LDH with PANI hybrid, charge transfer may occur between LDH and PANI. Figure S7 shows DOS of PANI calculated by Discrete Variational X (DV-X). From these curves, orbital of N 2p distributes widely within the band in the range from −10 to −3 eV for amino and from −8 to 0 eV for imino group, respectively. Such wideband structure may result in easy hopping for the charge transfer due to overlapping energy with both O 2p*_x_*, p*_y_* and p*_z_* in the LDH structure.

Based on the above results, the high specific capacitance and excellent cycling stability of CoAl LDH-PANI composite should be related to its well-designed nanosheets structure and synergistic effect between the inner LDH layer and outer PANI coating layer. They can be explained by the following reasons. (1) The CoAl LDH nanosheet can directly grow on the substrate with its ab-faces perpendicular to the substrate without any additive, which revealed a lower contact resistance and large specific surface area. 

**Figure 13:**
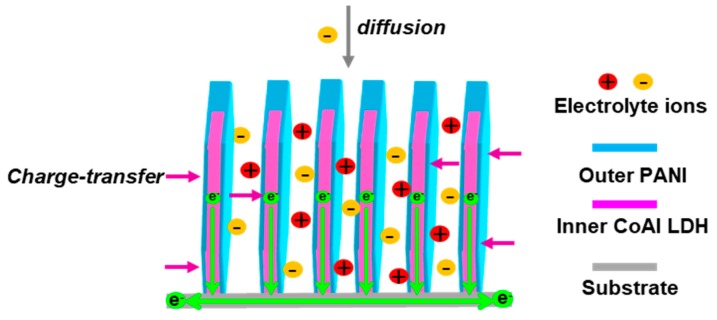
A schematic cross section illustration of electrolyte diffusion paths in CoAl LDH-PANI nanosheets.

As shown in Figure 13, the porous structures of CoAl LDH inner and thin PANI outer resulted in high utilization of active materials and short ionic diffusion path. (2) The space between CoAl LDH nanosheets is large enough to buffer the large volumetric swelling/shrinkage of PANI during charging/discharging process; while stable PANI shell also acts as a protection layer to preserve the CoAl LDH nanosheets from direct exposure to the corrosive environment. Especially Al can be dissolved in a strong alkaline solution. In this sense, the composite design that couple CoAl LDH with PANI can effectively make up the disadvantages of cycling stability of each component. (3) The enhancement in specific capacitance can be attributed to two aspects. The first aspect is due to the self-activation process of composites. At the initial cycle, active materials are not fully utilized, and it can delay the increase of the capacitance. After repeated charge-discharge process, the continuous activation of redox components is fully exposed to the electrolyte and they can greatly increase the capacitance by synergistic effect. The other aspect is the PANI can further maximize the specific capacitance of the whole electrode due to the decomposition of PANI to form new materials with highly electrochemically, and the improvement of ion diffusion rose the specific capacitance.

## 3. Materials and Methods 

### 3.1. Synthesis of the CoAl LDH Nanosheet Structures

The CoAl LDH was synthesized by a homogeneous hydrothermal method [9]. In a typical synthesis, Co(NO_3_)_2_·6H_2_O (0.857 mmol), Al(NO_3_)_3_·9H_2_O (0.286 mmol), NH_4_F (2.86 mmol) and urea (5.7 mmol) were dissolved in 50 mL distilled water. The mixed solution was stirred for 30 min and then transferred to an autoclave with a Teflon lining. A piece of clean nickel plate (50 mm × 10 mm × 0.1 mm) was immersed into the reaction solution. The autoclave was sealed and heated at 120 °C for 6 h, subsequently cooled to room temperature. Finally, the sample was taken out gently from the solution and washed three times with distilled water. The sample was then dried at room temperature. The load weight of CoAl LDH on the Ni substrate was measured with an analytical microbalance, and the mass loading was about 1.4 mg/cm^2^.

### 3.2. Synthesis of the CoAl LDH-PANI Nanocomposites

Hybridization with polymer PANI was carried out by a potentiostatic electrodeposition technique in an aqueous medium (0.05 M aniline + 0.02 M sodium dodecyl sulfate + 0.1 M LiClO_4_). Cyclic voltammetry experiments were performed to determine the deposition potential of PANI in a three-electrode electrochemical cell with a Ni substrate as the working electrode, a saturated calomel electrode (SCE) as the reference electrode, and a Pt plate as the counter electrode, respectively [23]. The optimum redox potential is selected at 1.18 V vs. SCE electrode according to the CV curve of aniline monomer (Figure S1). Subsequently, the Ni substrate/CoAl LDH film was used as the working electrode for the deposition of PANI. The deposition time varied from 50 to 600 s. After deposition, extreme caution must be taken when the as-prepared CoAl LDH-PANI electrode was washed with distilled water to prevent wash away PANI coating. And then the composite electrode was dried at room temperature. Finally, the mass loading of these deposited PANI on the Ni plate was obtained by the weight difference before and after electrochemical deposition.

### 3.3. Structural Characterization, Theoretical Calculations and Electrochemical Performance Measurements

The chemical structures of the samples were examined by Fourier transform infrared spectrometer (FTIR, FTIR4100, JASCO Corp.,Tokyo, Japan) analysis with ATR attachment. X-ray diffraction (XRD) patterns of the samples were obtained on an X-ray diffractometer with monochromated Cu Kα radiation (RINT-2100, Rigaku Corp., Tokyo, Japan). Synchrotron X-ray diffraction (SXRD) measurements in this study were performed at SPring-8, BL02B2 (Hyogo, Japan). The surface morphologies of the samples were characterized by field emission scanning electron microscopy (FESEM, JSM-6500F, JEOL, Ltd., Tokyo, Japan) equipped with EDX analyzer. The surface compositions of the samples were analyzed by X-ray photoelectron spectroscopy (XPS, AXIS Ultra DLD, Kratos Analytical Ltd., Manchester, UK). The band structure and density of states (DOS) curves of the host CoAl LDH crystals were calculated by the Vienna ab-initio simulation with VASP 5.3 [54,55]. The model structure of the calculation was the LDH with Co: Al of 3:1 with intercalation of chlorine anion. The structure was optimized up to the convergence and then the band structure was calculated. These calculations were carried out by Perdew-Burke-Ernzerhof (PBE) potentials. The DOS curves for guest PANI molecules in the supporting information (Figure S7) were calculated by DV-Xα simulation [56].

The electrochemical measurements were carried out in a three-electrode mode using the HZ 7000 electrochemical workstation (Hokuto Denko Corp., Tokyo, Japan) at room temperature with the 1 M KOH/KCl aqueous solution. Cyclic voltammetry (CV) and galvanostatic charge-discharge (GCD) were measured in a three-electrode cell within the potential window between 0 to 0.56 V. The Ni substrate/sample was used as the working electrode, an SCE as the reference electrode, and a Pt plate as the counter electrode. Electrochemical impedence spectroscopy (EIS) measurements were conducted by using an AC voltage with 5 mV amplitude at 0.3 V in a frequency range from 0.01 to 100 kHz. 

The specific capacitance and specific capacity values were calculated from the CV curves using the following Equations (2) and (3) [57]:

(2) $$Capacitance_{sp}=\frac{1}{sm\left(V_{h}-V_{l}\right)}\overset{V_{h}}{\underset{V_{l}}{\int }}idV$$

(3) $$Capacity_{sp}=\frac{1}{3.6\times sm}\overset{V_{h}}{\underset{V_{l}}{\int }}idV$$

where *C* (F/g) and *C* (mAh/g) represent the specific capacitance and specific capacity from the CV curves; *s* and *m* are scanning rate and mass of the samples separately; $V_{h}$ and $V_{l}$ represent high and low potential values; *i* is the oxidation or reduction current. Specific capacitance and specific capacity were also calculated from the galvanostatic charge-discharge curves, by Equations (4) and (5) [58]:

(4) $$Capacitance_{sp}=\frac{2i_{m}\int Vdt}{\left(V_{h}^{2}-V_{l}^{2}\right)}$$

(5) $$Capacity_{sp}=\frac{2i_{m}\int Vdt}{3.6\times \left(V_{h}+V_{l}\right)}$$

where *C* (F/g) and *C* (mAh/g) represent the specific capacitance and specific capacity from the GCD curves; *i_m_* is the current density; ∫Vdt is the integral current area; $V_{h}$ and $V_{l}$ represent high and low potential values.

## 4. Conclusions

In summary, we have successfully used in hydrothermal-electrodeposition method to prepare a novel inner/outer layer structural CoAl LDH-PANI nanocomposite in which the CoAl LDH nanofilms are well grown on Ni surface and the inner CoAl LDH is decorated by outer PANI layer. The hybrid architecture CoAl LDH-PANI exhibited greatly enhanced specific capacitance and cyclic stability (528 F/g at a current density of 10 A/g, 142.7% retention after 6000 cycles) and was superior to the non-decorated CoAl LDH (425 F/g at a current density of 10 A/g, 83.3% retention after 6000 cycles). The hydrothermal-electrodeposition method for synthesis of LDH-PANI growth on Ni substrate was straightforward and controllable. Our study results indicate that the PANI can be oxidized to other products which can act in the alkaline solution to increase of the capacitance under a proper working electrode and device voltage. Future studies are required to further identify the specific molecular structures of PANI degraded active products for the enhanced electrochemical activity of the LDH-based hybrid capacitor, and they are currently underway to examine in our laboratory. The possible mechanism of synergistic effect is also proposed in a new perspective. We deem that our research will be valuable in improving knowledge about LDHs/conducting polymer hybrid. The novel design strategy presented here also have a potential application for the direct design and fabrication of other hydroxides/oxides and conductive polymers hybrid films, for obtaining high electrochemical performance supercapacitor electrode material.

## Supplementary Materials

The Supplementary Materials are available online.
