# Supplementary material for: Enhanced Supercapacitor Performance Based on CoAl Layered Double Hydroxide-Polyaniline Hybrid Electrodes Manufactured Using Hydrothermal-Electrodeposition Technology
Source: Molecules. 2019 Mar 10;24(5):976. doi: 10.3390/molecules24050976 (PMC6429460; doi:10.3390/molecules24050976)
Supplement: Supplementary file 1 [file molecules-24-00976-s001.pdf]

molecules

MDPI

Supplementary Material

Enhanced Supercapacitor Performance Based on CoAl Layered Double Hydroxide-Polyaniline Hybrid Electrodes Manufactured Using Hydrothermal-Electrodeposition Technology

Guoshen Yang, Takahiro Takei *, Sayaka Yanagida and Nobuhiro Kumada

Center for Crystal Science and Technology, University of Yamanashi, 7-32 Miyamae, Kofu, Yamanashi 400-8511, Japan; g16dga02@yamanashi.ac.jp (G.Y.); syanagida@yamanashi.ac.jp (S.Y.); kumada@yamanashi.ac.jp (N.K.)

* Correspondence: takei@yamanashi.ac.jp; Tel.: +81-55-220-8616

Calculation of the electrochemically active Co (II) amount in both the pristine CoAl LDH and CoAl LDH-PANI electrodes

For the pristine CoAl LDH and CoAl LDH-PANI electrodes, the oxidation and reduction reactions could be explained by the following Equation (1), and the theoretical specific capacitance (C_t) could be calculated by the following Equation (2):

$$\mathrm{Co(OH)_2 + OH^- \leftrightarrow CoOOH + H_2O + e^-} \quad (1)$$

$$C_t = \frac{nF}{\Delta EM} \quad (2)$$

where ΔE is the potential range; M is the molecular mass of monomer; F is the Faraday constant (96485 C/mol); n is the average number of electrons transferred during the redox reaction.

The theoretical capacitance value of ~ 1834 F/g is obtained in the potential range of 0 ~ 0.56 V for $Co(OH)_2$. Therefore, we can simply calculate the electrochemically active Co (II) amount in both the pristine CoAl LDH and composite electrodes. For the CoAl LDH electrode, the practical specific capacitance value of ~ 525 F/g is calculated from Figure 6 (main paper), and its electrochemically active Co (II) amount is about 28.6%. If we assume that there is no interaction between CoAl LDH and PANI in the composite, the specific capacitance of CoAl LDH-PANI should be the sum of specific capacitance of CoAl LDH and PANI. For the CoAl LDH-PANI (PANI: CoAl LDH mass ratio of 0.21: 1), its specific capacitance can be calculated by the following Equation (3):

$$C_{LDH-PANI} = C_{LDH}\frac{1}{(1+0.21)} + C_{PANI}\frac{0.21}{(1+0.21)} \quad (3)$$

Based on the CV curves in Figure 6a (main paper), the practical specific capacitances of CoAl LDH-PANI and PANI are 318 and 120 F/g. After calculation by Equation (3), the specific capacitance of CoAl LDH in the composite is 360 F/g. The electrochemically active Co (II) amount of CoAl LDH in composite is about 19.6%. Compare the electrochemically active Co (II) amount in the both pristine CoAl LDH and CoAl LDH-PANI electrodes, it indicates that the inner active CoAl LDH in the composite have not been fully utilized.

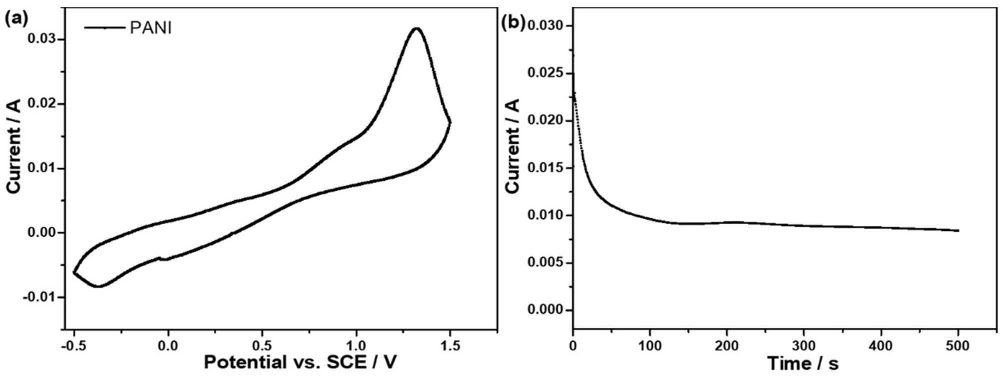

Figure S1. (a) CV of 0.05 M aniline in an aqueous solution (0.02 M SDS + 0.1 M $LiClO_4$) on the Ni plate at a scan rate of 20 mV/s. An anodic peak appears at ~1.18 V, which corresponds to the electropolymerization of aniline monomer. (b) Chronoamperograms of 0.05 M aniline in an aqueous solution (0.02 M SDS + 0.1 M $LiClO_4$) on the Ni plate at 1.18 V versus SCE.

The electrochemical polymerization technique was used to prepare the PANI deposition on CoAl LDH surface. CV experiments were performed to determine the deposition potential of PANI in a three-electrode cell. As shown in Figure S1a, the optimum deposition potential of the PANI film is fixed at 1.18 V vs. SCE according to the CV curve. Then the potentiostatic electrodeposition technique is used to prepare electrodeposition polyaniline on CoAl LDH surface at 1.18 V versus SCE, as seen from Figure S1b.

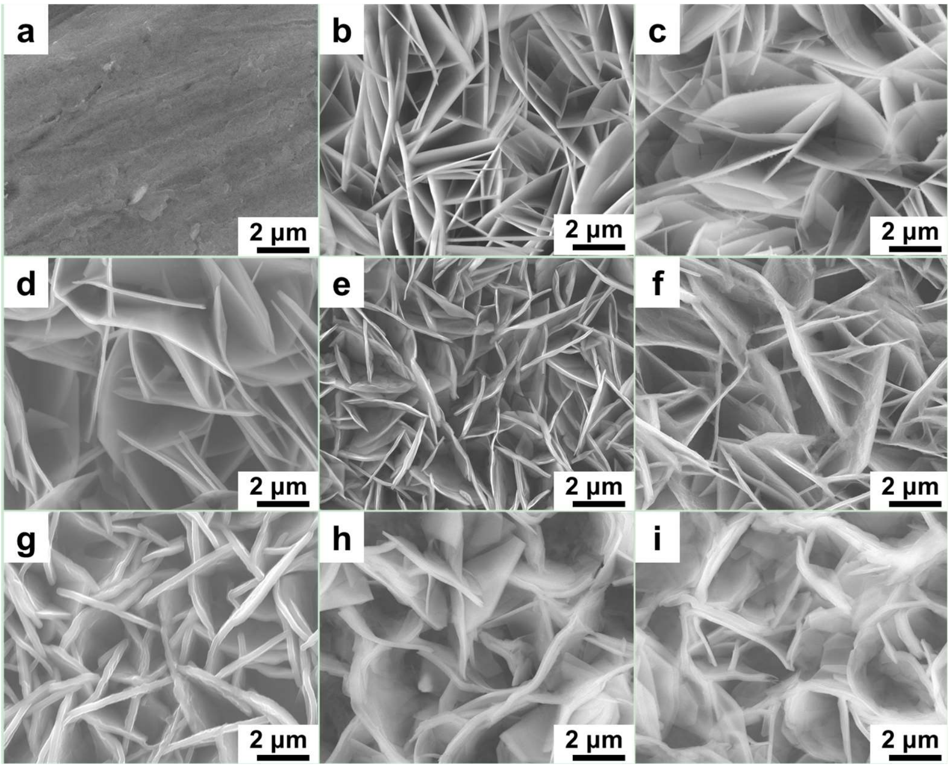

Figure S2. (a-i) FESEM micrographs of (a) PANI obtained by electrodeposition 600 s, (b-i) the CoAl LDH-PANI obtained by electrodeposition for various times: (b) 0 s, (c) 50 s, (d) 100 s, (e) 200 s, (f) 300 s, (g) 400 s, (h) 500 s, and (i) 600 s.

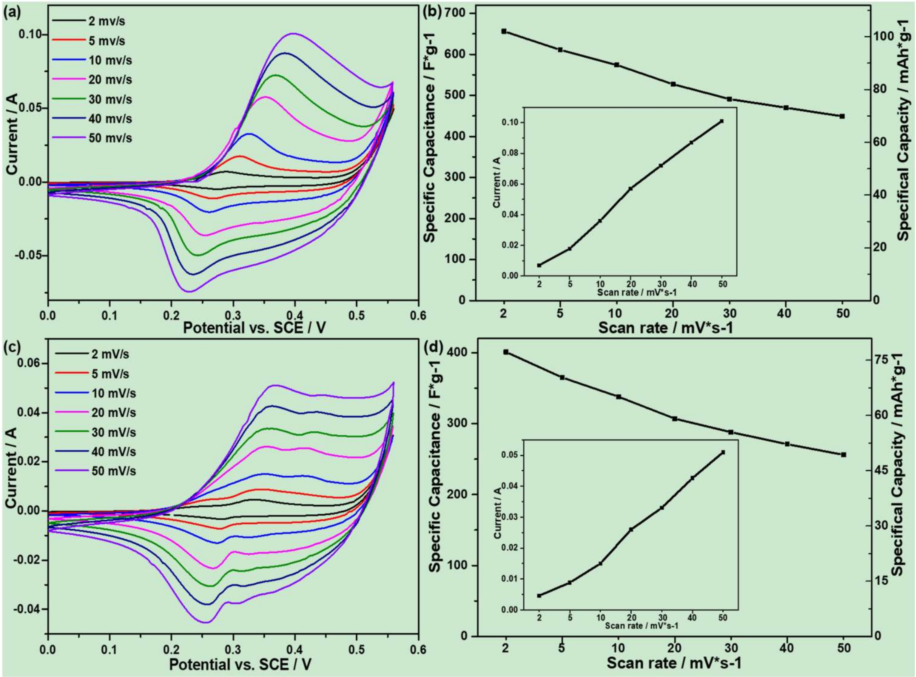

Figure S3. (a, c) CV curves of CoAl LDH and CoAl LDH-PANI electrodes at various current densities. (b, d) Specific capacitance and specific capacity of CoAl LDH and CoAl LDH-PANI electrodes calculated from CV curves. The insets in Figure S3b, d show the linearity of anodic current density with scan rate.

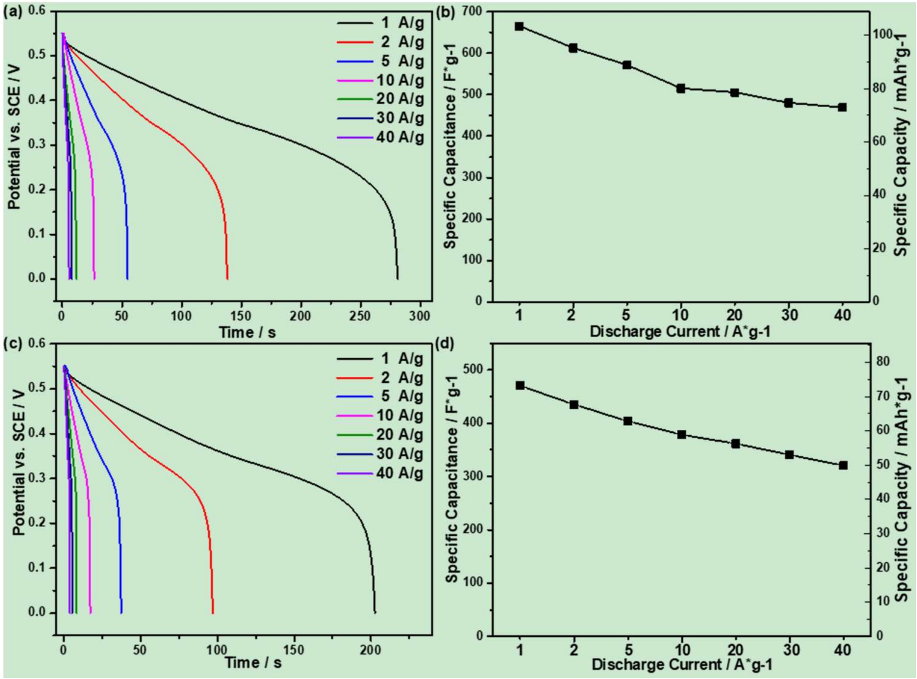

Figure S4. (a, c) Galvanostatic discharge curves of CoAl LDH and CoAl LDH-PANI electrodes at various current densities. (b, d) Specific capacitance and specific capacity of CoAl LDH and CoAl LDH-PANI electrodes calculated from galvanostatic discharge curves.

Figure S4a,c show galvanostatic discharge curves of the pristine CoAl LDH and CoAl LDH-PANI at different current densities. As shown in Figure S4b, the pristine CoAl LDH shows a specific capacitance of ~ 665 F/g (103.4 mAh/g) at a current density of 1 A/g and ~ 469 F/g (73 mAh/g) at 40 A/g, with a loss of 29.5% at 40 A/g. In comparison, the CoAl LDH-PANI in Figure S4d shows a specific capacitance of ~ 470 F/g (73.2 mAh/g) at a current density of 1 A/g and ~ 318 F/g (49.9 mAh/g) at 40 A/g, with a loss of 32.3% at 40 A/g. The GCD test results indicated that although the specific capacitance of CoAl LDH was greater than CoAl LDH PANI, the CoAl LDH-PANI still has the good rate capability.

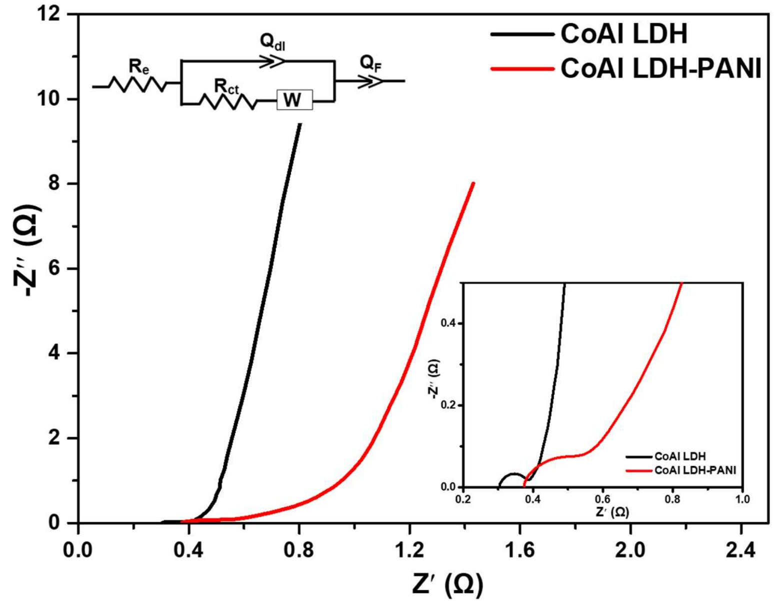

Figure S5. Nyquist plots of the EIS in the frequency range from 0.01 Hz to 100 kHz with an AC amplitude of 5 mV in 1 M KOH/KCl electrolyte.

The inset shows the equivalent circuit diagram. R_e is the equivalent series resistance, including the ionic resistance of the electrolyte, the intrinsic resistance of the substrate and electroactive material, and contact resistance at the interface between electrolyte and electrode [1]. R_{ct} is the charge-transfer resistance. Due to the porous surface of electrodes, the constant phase element (CPE) is used in the equivalent circuit. The impedance of CPE is defined as $Q = \frac{1}{T(jw)^n}$, where T and n are the frequency-independent constant and a correction factor range from -1 to 1. If n=-1, the CPE is an inductor and if n=1, CPE is a pure capacitor and if n=0, CPE is a pure resistor [2]. Q_{dl} represents the constant phase element, which is mainly attributed to the double-layer capacitance. Q_F represents the constant phase element, which is mainly attributed to the faradic pseudocapacitance. W is the Warburg resistance, which is caused by the diffusion/transport of OH^- ions within the porous structure of electrodes [3].

Table 1. The R_e, R_{ct}, W and n parameters of electrodes obtained from the Nyquist plot fitting.

Electrode	R_e (Ω)	R_{ct} (Ω)	W (Ω/s$^{1/2}$)	n_{dl}	n_F
CoAl LDH	0.304	0.076	0.137	0.891	1
CoAl LDH-PANI	0.375	0.381	0.315	0.729	0.974

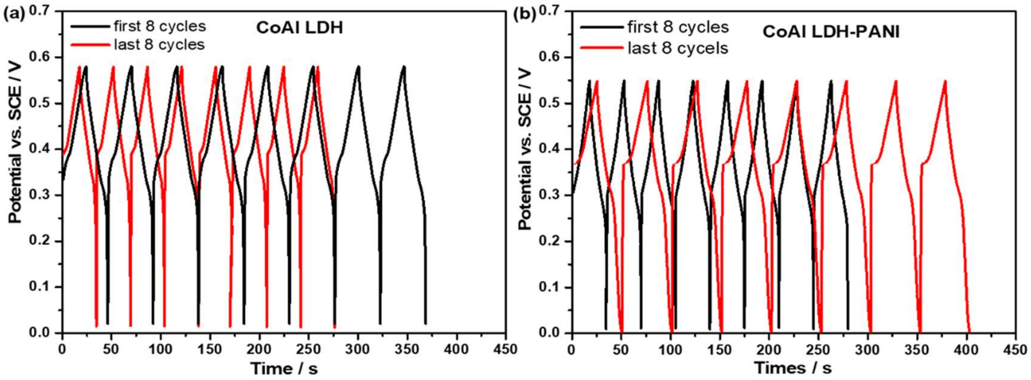

Figure S6. Galvanostatic charge-discharge curves of first 8 cycles and last 8 cycles for (a) CoAl LDH and (b) CoAl LDH-PANI during 6000 GCD cycles (current density of 10 A/g).

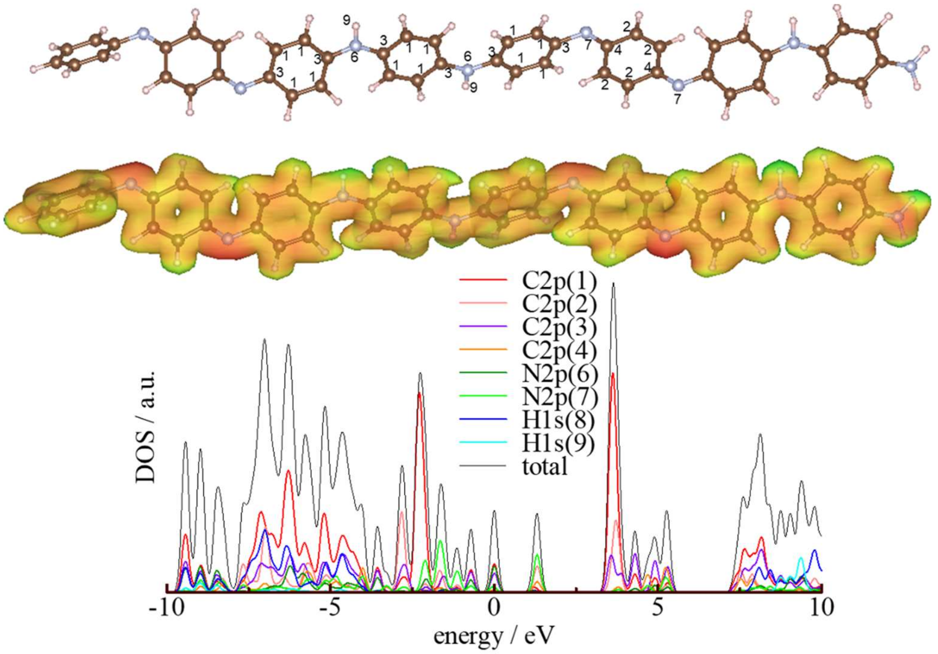

Figure S7. Charge density model and DOS of PANI molecules.

$$4OH^- \leftrightarrow O_2^- + 2H_2O + 3e^- \qquad E_0 = 0.645\ V \qquad (1)$$

Superoxide ions O_2^- have strong oxidizing properties, it can be much more stable in alkaline solution (Equation 1) [4, 5]. Based on the analysis of the FTIR and XPS of before and after 6000 cycles GCD test, we deemed the oxidative degradation of PANI in alkaline solution. Based on the analysis results of FTIR and XPS (Figure 9 and Figure 11 in main paper), we proposed that the possible degradation process is as follows. First, for the CoAl LDH-PANI electrode, the de-doping of SDS of the PANI backbone occurs in the alkaline solutions (Scheme S1) [6]. Then the PANI shell of CoAl LDH-PANI composite further degradation occurs in alkaline solution and the high potential. During the long-term GCD cycling test, superoxide ions are easily generated simultaneously at high potentials. The strong oxidation of superoxide ions can further oxidative degradation of PANI. The terminal group NO_2 may be due to the existence of superoxide ions to oxidize amino group and imino group to form.

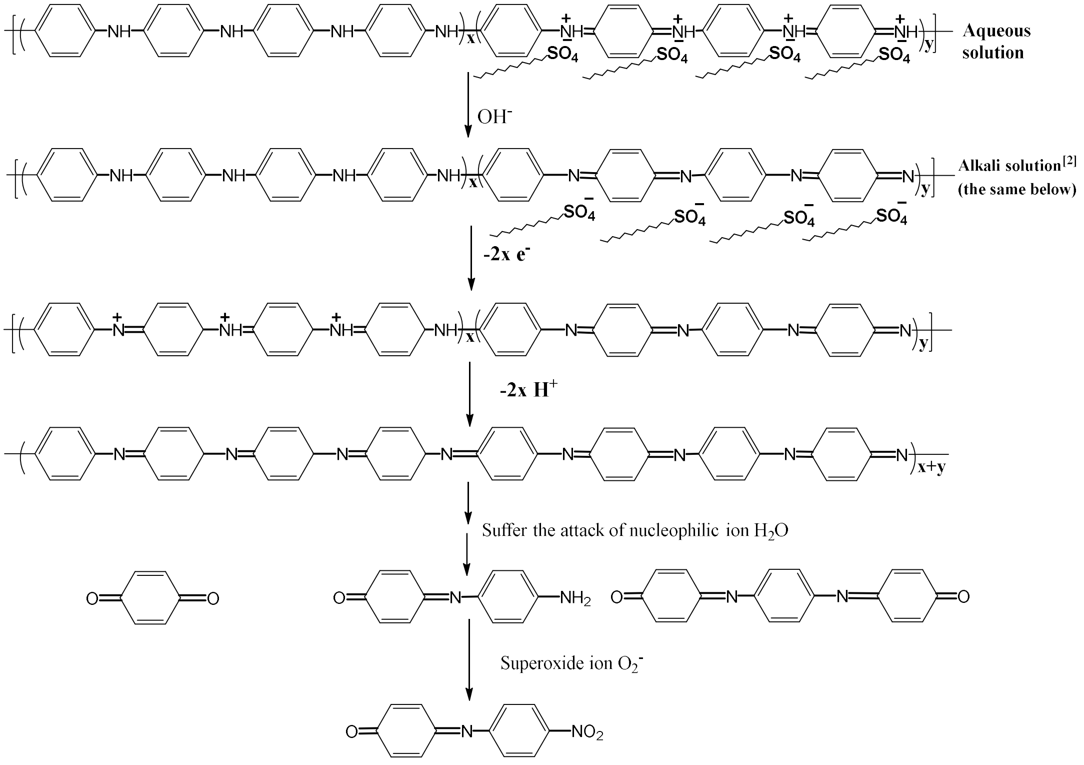

Scheme S1. Degradation of PANI during electrochemical process in the alkaline solutions.

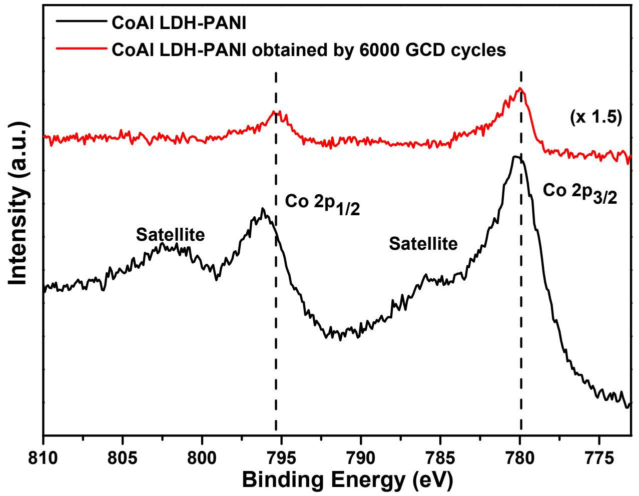

Figure S8. Co 2p spectra of pristine CoAl LDH-PANI and CoAl LDH-PANI obtained by 6000 GCD cycles.

References

1. Fang, J.; Li, M.; Li, Q. Microwave-assisted synthesis of CoAl-layered double hydroxide/graphene oxide composite and its application in supercapacitors. *Electrochim. Acta* **2012**, *85*, 248-255.
2. Zhou, Y.; Xu, H.; Lachman, N. Advanced asymmetric supercapacitor based on conducting polymer and aligned carbon nanotubes with controlled nanomorphology. *Nano Energy* **2014**, *9*, 176-185.

3. Masikhwa, T.M.; Dangbegnon, J.K.; Bello, A. Preparation and electrochemical investigation of the cobalt hydroxide carbonate/activated carbon nanocomposite for supercapacitor applications. *J. Phys. Chem. Solids* **2016**, *88*, 60-67.
4. Bard, A.J.; Parsons, R.; Jordan, J. *Standard potentials in aqueous solution*. IUPAC-Marcel Dekker Inc.: New York, NY, USA, **1985**; pp. 61-62.
5. Hayyan, M.; Hashim, M.A.; AlNashef, I.M. Superoxide ion: generation and chemical implications. *Chem. Rev.* **2016**, *116*, 3029-3085.
6. Leng, W.; Zhou, S.; Gu, G.; Wu, L. Wettability switching of SDS-doped polyaniline from hydrophobic to hydrophilic induced by alkaline/reduction reactions. *Colloid Interface Sci*. **2012**, *369*, 411-418.
